# Draft Genome Sequence of an Isolate of Nontypeable Haemophilus influenzae from an Acute Exacerbation of Chronic Obstructive Pulmonary Disease in Tasmania

**DOI:** 10.1128/MRA.00375-20

**Published:** 2020-05-07

**Authors:** Rajendra KC, Kelvin W. C. Leong, Belinda McEwan, Julia Lachowicz, Nicholas M. Harkness, Steve Petrovski, Gunasegaran Karupiah, Ronan F. O’Toole

**Affiliations:** a Tasmanian School of Medicine, College of Health and Medicine, University of Tasmania, Hobart, Tasmania, Australia; b Department of Pharmacy and Biomedical Sciences, School of Molecular Sciences, College of Science, Health, and Engineering, La Trobe University, Melbourne, Victoria, Australia; c Department of Microbiology and Infectious Diseases, Royal Hobart Hospital, Hobart, Tasmania, Australia; d Department of Respiratory and Sleep Medicine, Royal Hobart Hospital, Hobart, Tasmania, Australia; e Department of Physiology, Anatomy and Microbiology, School of Life Sciences, La Trobe University, Melbourne, Victoria, Australia; f Department of Clinical Microbiology, School of Medicine, Trinity College Dublin, Dublin, Ireland; University of Rochester School of Medicine and Dentistry

## Abstract

Nontypeable Haemophilus influenzae (NTHi) is an important cause of human illness, including pneumonia and acute exacerbations of chronic obstructive pulmonary disease (COPD). We report here the draft genome of an isolate of NTHi collected from the sputum of a patient presenting with COPD in Tasmania, Australia.

## ANNOUNCEMENT

Chronic obstructive pulmonary disease (COPD) is a serious, progressive condition characterized by a persistent reduction in lung airflow (1). It has emerged as the third leading cause of mortality, claiming more than 3 million lives worldwide in 2016 (2). In Australia, it is estimated that COPD affects 1.45 million people (3). COPD is an important disease in the state of Tasmania, where higher rates of smoking are observed with respect to the national rate (4). Nontypeable Haemophilus influenzae (NTHi) is a key pathogen that colonizes damaged airways in COPD patients and causes acute exacerbations that contribute to morbidity and mortality (5–7). Here, we present the draft assembled genome sequence of an NTHi strain isolated from a case of COPD in Tasmania.

NTHi strain RHH-38 was isolated in 2018 at the Royal Hobart Hospital in Tasmania from the sputum of a COPD patient presenting with an acute exacerbation. The sputum specimen was homogenized and cultured on chocolate blood agar plates at 35°C in a CO_2_ atmosphere followed by storage at 2 to 8°C. Bacterial identification was performed using a Bruker matrix-assisted laser desorption ionization–time of flight (MALDI-TOF) mass spectrometer. A single colony of Haemophilus influenzae was suspended in 200 μl phosphate-buffered saline (PBS), and then genomic DNA was extracted using the DNeasy blood and tissue kit (catalog number 69504; Qiagen, USA). The genomic DNA preparation was further purified using the High Pure PCR template preparation kit (catalog number 11796828001; Roche, Germany). DNA library preparation was carried out using a Nextera XT DNA library preparation kit (catalog number FC-131-1024; Illumina, USA) as described previously (8, 9). Sequencing was performed using an Illumina MiSeq platform with 150-bp paired-end sequencing. In total, 1,161,034 paired-end reads were generated, representing an average read depth of 88.83-fold. Reads were trimmed of adapters using Trimmomatic (10), and *de novo* assembly of reads was performed with SPAdes v3.12.0 (11). All parameters were set to default except for the size of k-mers, which were manually set to 21, 33, 43, 53, 63, and 75. This resulted in the generation of a 1,914,787-bp draft genome consisting of 68 contigs (≥200 bp) that covered 86.7% of the reference H. influenzae 86-028NP genome (12). The *N*_50_ value was 66,703 bp, and the overall GC content was 38.1%. The genome assembly quality, including completeness with respect to the reference genome, was determined using the QUAST quality assessment tool (13).

*In silico* MLST analysis, performed by submission of the draft genome to the H. influenzae multilocus sequence typing (MLST) website (https://pubmlst.org/hinfluenzae/) (14), assigned RHH-38 to sequence type 422 (ST422) based on seven housekeeping genes. The draft genome was annotated using RASTtk (15–17), which identified a total of 2,019 genes consisting of 1,960 coding sequences and 59 RNA genes. Default parameters were used for all software unless otherwise specified.

In conclusion, this study presents the published genome sequence assembly of an NTHi isolate from a case of COPD in Tasmania. The application of genome sequencing has the potential to provide insights into recurrent exacerbations of COPD due to NTHi and the ability to distinguish between relapse and reinfection.

This work was conducted in accordance with ethics approval number H0016214 from the Tasmanian Health and Medical Human Research Ethics Committee.

### Data availability.

This whole-genome shotgun project has been deposited at DDBJ/EMBL/GenBank under the accession number JAAECN000000000. The version described in this paper is version JAAECN010000000. The associated BioProject and BioSample accession numbers are PRJNA603840 and SAMN13942196, respectively.

## References

[1] Petsonk EL, Hnizdo E, Attfield M 2007 Definition of COPD GOLD stage I. Thorax 62:1107–1108. PMC2094288 18025144

[2] World Health Organization. 2017 Disease burden and mortality estimates: cause-specific mortality, 2000–2016. World Health Organization, Geneva, Switzerland http://www.who.int/healthinfo/global_burden_disease/estimates/en/.

[3] Toelle BG, Xuan W, Bird TE, Abramson MJ, Atkinson DN, Burton DL, James AL, Jenkins CR, Johns DP, Maguire GP, Musk AW, Walters EH, Wood-Baker R, Hunter ML, Graham BJ, Southwell PJ, Vollmer WM, Buist AS, Marks GB 2013 Respiratory symptoms and illness in older Australians: the Burden of Obstructive Lung Disease (BOLD) study. Med J Aust 198:144–148. doi:10.5694/mja11.11640. 23418694

[4] Australian Bureau of Statistics. 2019 Higher rates of chronic health conditions in Tasmania. Australian Bureau of Statistics https://www.abs.gov.au/ausstats/abs@.nsf/Lookup/by%20Subject/4364.0.55.001~2017-18~Media%20Release~Higher%20rates%20of%20chronic%20health%20conditions%20in%20Tasmania%20(Media%20Release)~10015.

[5] Sethi S, Murphy TF 2001 Bacterial infection in chronic obstructive pulmonary disease in 2000: a state-of-the-art review. Clin Microbiol Rev 14:336–363. doi:10.1128/CMR.14.2.336-363.2001. 11292642 PMC88978

[6] Sethi S, Evans N, Grant BJ, Murphy TF 2002 New strains of bacteria and exacerbations of chronic obstructive pulmonary disease. N Engl J Med 347:465–471. doi:10.1056/NEJMoa012561. 12181400

[7] Hillas G, Perlikos F, Tzanakis N 2016 Acute exacerbation of COPD: is it the “stroke of the lungs”? Int J Chron Obstruct Pulmon Dis 11:1579–1586. doi:10.2147/COPD.S106160. 27471380 PMC4948693

[8] Gautam SS, Kc R, Leong KW, Mac Aogáin M, O'Toole RF 2019 A step-by-step beginner’s protocol for whole genome sequencing of human bacterial pathogens. J Biol Methods 6:e110. doi:10.14440/jbm.2019.276. 31453259 PMC6706130

[9] Gautam SS, Mac Aogáin M, Cooley LA, Haug G, Fyfe JA, Globan M, O'Toole RF 2018 Molecular epidemiology of tuberculosis in Tasmania and genomic characterisation of its first known multi-drug resistant case. PLoS One 13:e0192351. doi:10.1371/journal.pone.0192351. 29466411 PMC5821347

[10] Bolger AM, Lohse M, Usadel B 2014 Trimmomatic: a flexible trimmer for Illumina sequence data. Bioinformatics 30:2114–2120. doi:10.1093/bioinformatics/btu170. 24695404 PMC4103590

[11] Bankevich A, Nurk S, Antipov D, Gurevich AA, Dvorkin M, Kulikov AS, Lesin VM, Nikolenko SI, Pham S, Prjibelski AD, Pyshkin AV, Sirotkin AV, Vyahhi N, Tesler G, Alekseyev MA, Pevzner PA 2012 SPAdes: a new genome assembly algorithm and its applications to single-cell sequencing. J Comput Biol 19:455–477. doi:10.1089/cmb.2012.0021. 22506599 PMC3342519

[12] Harrison A, Dyer DW, Gillaspy A, Ray WC, Mungur R, Carson MB, Zhong H, Gipson J, Gipson M, Johnson LS, Lewis L, Bakaletz LO, Munson RS Jr 2005 Genomic sequence of an otitis media isolate of nontypeable Haemophilus influenzae: comparative study with H. influenzae serotype d, strain KW20. J Bacteriol 187:4627–4636. doi:10.1128/JB.187.13.4627-4636.2005. 15968074 PMC1151754

[13] Gurevich A, Saveliev V, Vyahhi N, Tesler G 2013 QUAST: quality assessment tool for genome assemblies. Bioinformatics 29:1072–1075. doi:10.1093/bioinformatics/btt086. 23422339 PMC3624806

[14] Jolley KA, Bray JE, Maiden MCJ 2018 Open-access bacterial population genomics: BIGSdb software, the PubMLST.org website and their applications. Wellcome Open Res 3:124. doi:10.12688/wellcomeopenres.14826.1. 30345391 PMC6192448

[15] Aziz RK, Bartels D, Best AA, DeJongh M, Disz T, Edwards RA, Formsma K, Gerdes S, Glass EM, Kubal M, Meyer F, Olsen GJ, Olson R, Osterman AL, Overbeek RA, McNeil LK, Paarmann D, Paczian T, Parrello B, Pusch GD, Reich C, Stevens R, Vassieva O, Vonstein V, Wilke A, Zagnitko O 2008 The RAST server: Rapid Annotations using Subsystems Technology. BMC Genomics 9:75. doi:10.1186/1471-2164-9-75. 18261238 PMC2265698

[16] Brettin T, Davis JJ, Disz T, Edwards RA, Gerdes S, Olsen GJ, Olson R, Overbeek R, Parrello B, Pusch GD, Shukla M, Thomason JA III, Stevens R, Vonstein V, Wattam AR, Xia F 2015 RASTtk: a modular and extensible implementation of the RAST algorithm for building custom annotation pipelines and annotating batches of genomes. Sci Rep 5:8365. doi:10.1038/srep08365. 25666585 PMC4322359

[17] Overbeek R, Olson R, Pusch GD, Olsen GJ, Davis JJ, Disz T, Edwards RA, Gerdes S, Parrello B, Shukla M, Vonstein V, Wattam AR, Xia F, Stevens R 2014 The SEED and the Rapid Annotation of microbial genomes using Subsystems Technology (RAST). Nucleic Acids Res 42:D206–D214. doi:10.1093/nar/gkt1226. 24293654 PMC3965101

